# Retinoic Acid Promotes the In Vitro Growth, Patterning and Improves the Cellular Composition of Human Pluripotent Stem-Cell-Derived Intestinal Organoids

**DOI:** 10.3390/ijms23158624

**Published:** 2022-08-03

**Authors:** Na Qu, Braxton Jeffcoat, Pritiprasanna Maity, Rachael K. Christensen, Jorge O. Múnera

**Affiliations:** Department of Regenerative Medicine and Cell Biology, Medical University of South Carolina, Charleston, SC 29425, USA; qu@musc.edu (N.Q.); swooswansea98@gmail.com (B.J.); maity@musc.edu (P.M.); rkchris@g.clemson.edu (R.K.C.)

**Keywords:** human intestinal organoids, human pluripotent stem cells, midgut, hindgut, retinoic acid, patterning

## Abstract

Human intestinal organoids (HIOs) generated from human pluripotent stem cells hold great promise for modeling human development and as a possible source of tissue for transplantation. HIOs generate all of the main epithelial and mesenchymal cell types found in the developing human intestine and mature into intestinal tissue with crypts and villi following transplantation into immunocompromised mice. However, incomplete in vitro patterning and the presence of contaminating neurons could hinder their use for regenerative medicine in humans. Based on studies in model organisms, we hypothesized that the treatment of HIOs with all trans retinoic acid (ATRA) would improve their in vitro growth and patterning. We found that ATRA not only improved the patterning of HIOs, ATRA also increased organoid forming efficiency, improved epithelial growth, enriched intestinal subepithelial myofibroblasts (ISEMFs) and reduced neuronal contamination in HIOs. Taken together, our studies demonstrate how the manipulation of a single developmental signaling pathway can be used to improve the survival, patterning and cellular composition of HIOs.

## 1. Introduction

In the past decade, organoid models have revolutionized the study of the gastrointestinal (GI) epithelium [1,2,3,4]. Organoid models have provided significant insights into homeostasis and pathogenesis of GI organs. The most commonly used organoid models are derived from patient biopsy tissue and are grown as strictly epithelial structures. Since they are derived from patients, they will reflect the age and physiology of the patient. In contrast, human pluripotent stem cells can be differentiated into human intestinal and colonic organoids (HIOs and HCOs) which contain both epithelial and mesenchymal cell types [5,6]. Because they are generated through the sequential activation of developmental signals, early stages of human gut development are reflected.

Despite their utility for modeling early human intestinal development, HIOs are only partially patterned into proximal intestinal tissue in vitro. HIOs have been shown to express the duodenal marker PDX1 in vitro [7,8]. However, HIOs lack the in vitro expression of GATA4 [5,6], a transcription factor which is critical for the establishment of proximal intestinal identity [9,10]. Despite the lack of GATA4 expression in HIOs grown in vitro, HIOs transplanted into the kidney capsule of immunocompromised mice express GATA4 in their epithelium [5,7]. Recent single cell analyses of developing HIOs have revealed the presence of contaminating neural cells [11,12]. Therefore, current in vitro culture conditions lack the factors necessary to induce GATA4 expression in HIOs, resulting in incomplete patterning. Furthermore, current in vitro culture conditions do not limit the co-development of contaminating neural cells in HIOs.

Retinoic acid (RA) is a biologically active derivative of vitamin A. RA is synthesized from retinol (a form of Vitamin A found in foods), through a two-step process. First, retinol dehydrogenase-10 (RDH10) converts retinol to retinal. Subsequently, retinaldehyde dehydrogenase-2 (Raldh2) converts retinal to RA. Once RA is available, RA can bind to heterodimers of RA receptors (RARs) and retinoid X receptors (RXRs), known as RAR/RXR. In the absence of RA, RAR/RXRs recruit co-repressor complexes to Retinoic Acid Response Elements (RAREs), effectively inhibiting gene transcription. In the presence of RA, RA can bind to RAR/RXRs causing a conformational change resulting in the release of the co-repressor complexes and recruitment of co-activator complexes, resulting in the activation of gene transcription [13]. Studies in multiple model systems have described the effects of RA on the development and regeneration of the small intestine. Roles for RA in the induction of the expression of midgut markers [14,15,16], in the induction of cytodifferentiation [17] and in the induction of cell proliferation following resection [18], have all been described. Retinoic acid has also been shown to enhance enterocyte differentiation in murine small intestinal organoids [19] and enhance M-cell differentiation in human biopsy-derived intestinal organoids [20]. Based on this prior knowledge, we hypothesized that the treatment of HIOs with RA would improve their growth, patterning and cytodifferentiation. We found that RA treatment enhanced the organoid forming efficiency of HIOs. In addition, we found that retinoic acid improved epithelial growth and patterning. Furthermore, RA enriched intestinal subepithelial myofibroblasts (ISEMFs), improved niche factor expression and inhibited the co-development of neurons within organoids. Taken together, these results demonstrate how RA activation can improve the growth, cytodifferentiation and cellular fidelity of HIOs.

## 2. Results

### 2.1. Spheroid- and Clump-Derived HIOs Display Similar Transcriptomic Profiles

**Figure 1:**
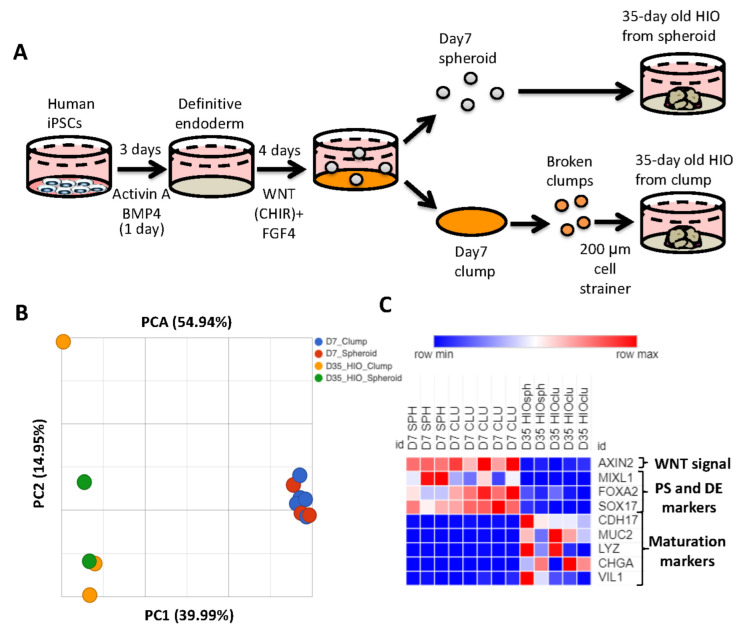
The source of mid/hindgut tissue does not affect HIO growth or maturation. (**A**) Schematic of mid/hindgut tissue generation and development of HIOs from spheroids or clumps. (**B**) Principal component analysis spheroid and clump derived mid/hindgut tissue and 35-day-old HIOs. (**C**) Heatmap of the WNT target gene AXIN, markers of primitive streak and maturation markers from TPM (transcripts per million) values.

Generation of HIOs from human pluripotent stem cells requires the successful execution of all steps during the differentiation process. One of the key roadblocks in the generation of HIOs is the generation of mid/hindgut spheroids from definitive endoderm. To bypass this roadblock, multiple groups have generated HIOs by passaging monolayers of mid-hindgut patterned definitive endoderm (DE) as clumps, and then plating these clumps in extracellular matrix. Over time, the cells within clumps will self-organize and develop into HIOs [21,22,23]. Although these methods have been reported to generate HIOs which resemble developing human intestine, a direct comparison of spheroid-generated and clump-generated HIOs has not been carried out previously. We therefore generated spheroids and clumps to generate HIOs (Figure 1A). We then performed RNA-seq analysis of 7-day-old spheroids and clumps, as well as 35-day-old HIOs grown from the former. Principal component analysis revealed that 7-day-old spheroids and clumps clustered together, while 35-day-old HIOs (from spheroids and from clumps) clustered together along principal component 1 (PC1) axis, which accounted for 40% of the cumulative variation among samples (Figure 1B). Closer examination of the RNA-seq data revealed that genes associated with WNT signaling (AXIN2), primitive streak (MIXL1) and definitive endoderm (FOXA2 and SOX17) were all highly enriched in 7-day-old spheroids/clumps compared to 35-day-old HIOs from spheroids/clumps (Figure 1C). In contrast, genes associated with maturation (CDH17) and cytodifferentiation (MUC2, LYZ, CHGA and VIL1) were all highly enriched in 35-day-old HIOs from spheroids/clumps compared to 7-day-old spheroids/clumps. These data indicate that clumps and spheroids share a similar transcriptional profile. Furthermore, our data indicate that the source of mid/hindgut tissue (whether spheroids or clumps) does not perturb the growth, maturation or cytodifferentiation of HIOs.

### 2.2. Retinoic Acid Improves HIO Outgrowth

**Figure 2:**
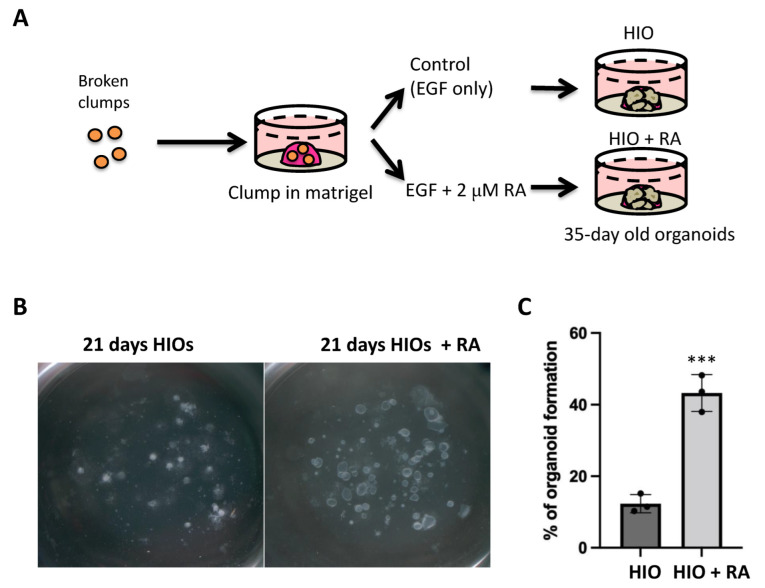
Retinoic acid treatment improves organoid generation. (**A**) Schematic of retinoic acid treatment experiments utilizing HIOs. (**B**) Stereomicroscope images of 21-day-old organoids from the treatment groups. (**C**) Quantification of organoid-forming efficiency. Individual point represents quantification from a single biological replicate (*n* = 3 per group). *** denotes *p* < 0.001 based on *t*-test.

The use of clumps to generate HIOs has several advantages including increased efficiency, increased applicability to IPSC lines that are not grown on extracellular matrix and it generates an increased amount of starting material with uniform size. Having established the similarity in gene expression profiles between spheroid and clump generated HIOs, we then tested our hypothesis that the treatment of HIOs with all trans retinoic acid (ATRA) would improve their in vitro patterning. Therefore, we treated one group of clump-derived HIOs with growth medium containing only EGF (herein referred to as HIO) as was carried out previously. In addition, we treated one group with HIO growth medium supplemented with 2 µM RA (herein referred to as HIO + RA). We then grew the respective organoids from day 7 to day 35 in their respective medium (Figure 2A). We observed that the number and size of organoids growing in the HIO + RA group was significantly increased in RA-treated HIOs (Figure 2B). To quantify the results of these observations, we plated clumps at low density (50–100 clumps per well) in Matrigel and counted them. We then counted the number of organoids that grew from these clumps at day 21. This quantification revealed that HIOs treated with RA had a 3-fold higher organoid-forming efficiency than the control HIOs (Figure 2C). These data indicate that RA promotes organoid formation from mid/hindgut clumps.

### 2.3. Retinoic Acid Improves the Patterning of HIOs

**Figure 3:**
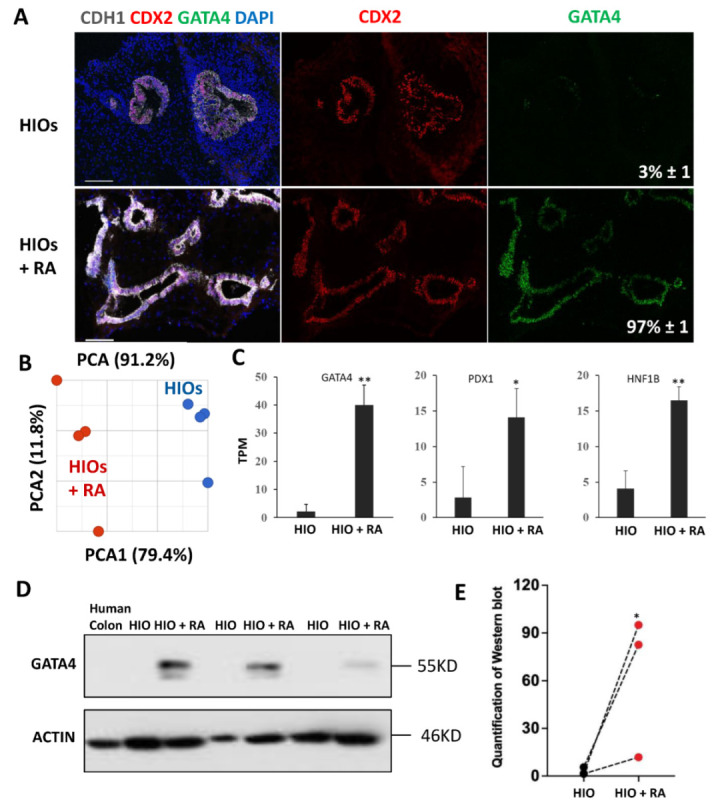
Retinoic acid patterns of HIOs in vitro. (**A**) Immunofluorescence staining of 35-day-old organoids from (HIOs and HIOs + RA) for CDH1 (white), CDX2 (red), GATA4 (green) and counterstained with DAPI. Numbers in GATA4 panel represent the percentage of GATA4-positive epithelial cells ± the SEM. N = 7 organoids per treatment group. (**B**) Principal of 35-day-old HIOs and HIOs + RA. N = 4 per treatment group. (**C**) Graph of TPM values from RNA-seq data of 35-day-old HIOs and HIOA. Values are displayed as the mean ± SD. n = 4 per treatment group. (**D**) Western blot for GATA4 expression from human colon (negative control), HIOs and HIOA from 3 different experiments. (**E**) Densitometric analysis of Western blot data from the panel (**C**). * denotes a *p* value of <0.05. ** denotes a *p* value of <0.01 based on *t*-test.

HIOs transplanted into the kidney capsule of immunocompromised mice express the proximal intestinal transcription factor GATA4. GATA4 has be shown to play a key role in maintaining proximal intestinal identity [9,10]. RA has been shown to induce GATA4 expression in non-intestinal cells [24]. Therefore, we performed immunofluorescence staining for GATA4 on organoids, to determine if RA could activate GATA4 expression. These experiments revealed that 35-day-old HIOs lacked GATA4 expression while 35-day-old HIOs + RA expressed GATA4 (Figure 3A). Image quantification revealed that, while only 3% of the epithelial cells in 35-day-old HIOs expressed GATA4, >97% of the epithelial cells in 35-day-old HIOs + RA expressed GATA4 (Figure 3A). These results indicate that RA improves organoid patterning. In order to broadly interrogate the regional identity and maturation of HIOs and HIOs + RA, we performed RNA-seq analysis of HIOs and HIOs + RA. Principal component analysis revealed that 35-day-old HIOs and 35-day-old HIOs + RA clustered together (based on treatment) along principal component 1 (PC1) axis, which accounted for 79.4% of the cumulative variation among samples (Figure 3B). Examination of GATA4, PDX1 and HNF1B mRNA expression levels from RNA-seq data revealed that all these genes had significantly increased expression in HIOs + RA compared to HIOs (Figure 3C). We further confirmed the increase in GATA4 expression by Western blot (Figure 3D,E). In summation, these results support the conclusion that RA improves the in vitro patterning of HIOs.

### 2.4. Retinoic Acid Promotes Epithelial Growth and Enriches ISEMFs in HIOs

**Figure 4:**
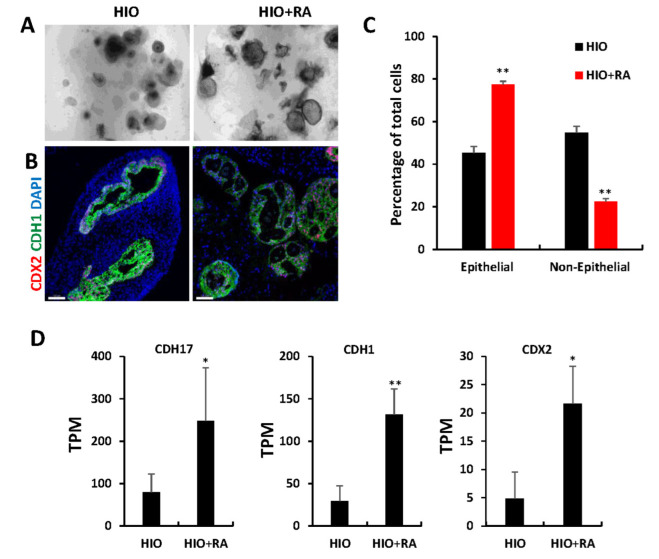
Retinoic acid promotes epithelial growth. (**A**) Light microscopy images of HIOs and HIOs + RA. (**B**) Immunofluorescence staining of 35-day-old organoids from (HIOs and HIOs + RA) for CDX2 (red), CDH1 (green) and counterstained with DAPI. (**C**) Quantification of the percentage of epithelial and non-epithelial cells per organoid section. Data are presented as the mean ± SEM. n = 7–8 organoids per treatment group. (**D**) Graphs of TPM values of epithelial genes from RNA-seq data of 35-day-old HIOs and HIOs + RA. Values are displayed as the mean ± SD. n = 4 per treatment group. * denotes a *p* value < 0.05. ** denotes a *p* value of <0.01 based on *t*-test.

The examination of organoids through light microscopy revealed that after 35 days, HIOs + RA continued to have improved epithelial growth and less mesenchyme than HIOs grown in control media (Figure 4A). Immunofluorescence staining and image quantification of organoids stained for the pan-intestinal epithelial marker CDX2, the epithelial marker CDH1 (also known as E-cadherin) and DAPI revealed a significant increase in the percentage of epithelial cells within HIOs treated with RA compared to control HIOs (Figure 4B,C). Gene ontology analysis of the “Cellular component” category revealed a significant increase in genes associated with cell–cell junction, apical part of cell, microvillus (Table S1), consistent with an increase in epithelium in RA-treated HIOs. To further confirm these observations, we examined the RNA-seq data from 35-day-old HIOs and HIOs + RA. Cadherin-17 (CDH17), CDH1 and Caudal Type Homeobox 2 (CDX2) mRNA levels were significantly increased in HIOs treated with RA compared to control HIOs. These results indicate that the epithelial composition of HIOs is improved by RA treatment.

**Figure 5:**
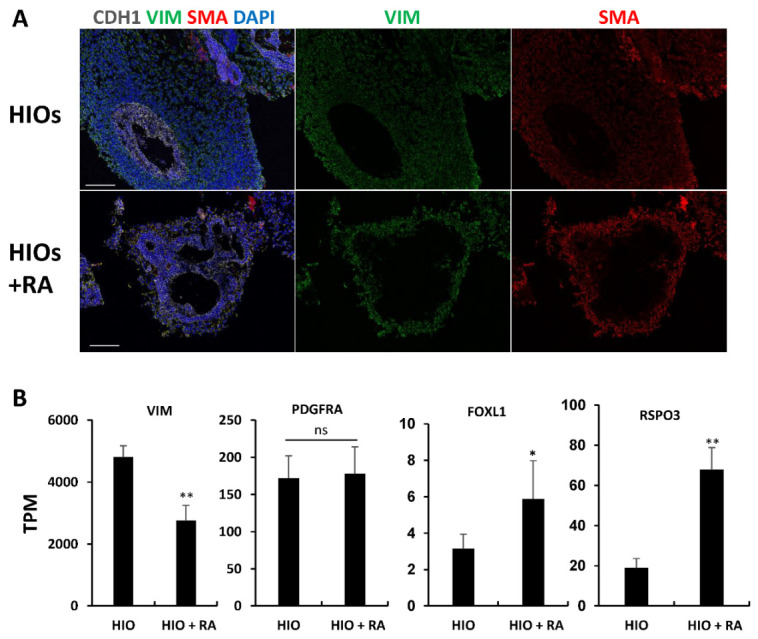
Retinoic acid enriches epithelium-supportive mesenchyme. (**A**) Immunofluorescence staining of 35-day-old organoids from (HIOs and HIOs + RA) for CDH1(white), VIM (green), SMA (red) and counterstained with DAPI. (**B**) Graphs of TPM values of epithelial genes from RNA-seq data of 35-day-old HIOs and HIOs + RA. Values are displayed as the mean ± SD. n = 4 per treatment group. * denotes a *p* value < 0.05. ** denotes a *p* value < 0.01 based on *t*-test.

HIOs and HIOs + RA are grown in media-containing EGF, but lack other factors required for epithelial growth such as NOGGIN and R-SPONDIN. We postulated that despite having less mesenchyme, RA-treated organoids would have an increased proportion of epithelium-supportive mesenchyme. To test this, we performed immunofluorescence staining for vimentin (VIM) and smooth muscle actin (SMA). Co-staining of VIM and SMA in mesenchymal cells has been used to identify intestinal subepithelial myofibroblasts (ISEMFs) which can support the growth of human intestinal epithelium [25,26,27]. We found that HIOs only had rare VIM+/SMA+ cells. In contrast, HIOs + RA had VIM+/SMA+ cells which surrounded the epithelium (Figure 5A). To further confirm differences in the mesenchyme of HIOs and HIOs + RA, we examined our RNA-seq data (Figure 5B). Examination of the mesenchymal marker Vimentin (VIM) revealed a significant increase in mRNA expression in HIOs compared to HIOs + RA. This is consistent with our immunostaining results and quantification of epithelial and non-epithelial cells. Interestingly, despite an overall increase in the amount of non-epithelial cells in HIOs compared HIOs + RA, the expression of PDGFRA, a marker of mesenchymal cells which directly interact with the epithelium [28,29], was not different between the two groups. Furthermore, the mRNA expression levels of makers of intestinal epithelial stem cell niche supportive mesenchyme, FOXL1 and RSPO3, were higher in HIOs + RA than in the control HIOs. These data indicate that although RA restricts the growth of mesenchyme, RA seems to select mesenchymal cells which can support the growth and self-renewal of stem cells within the organoid epithelium.

### 2.5. Retinoic Acid Inhibits the Co-Development of Neurons in HIOs

**Figure 6:**
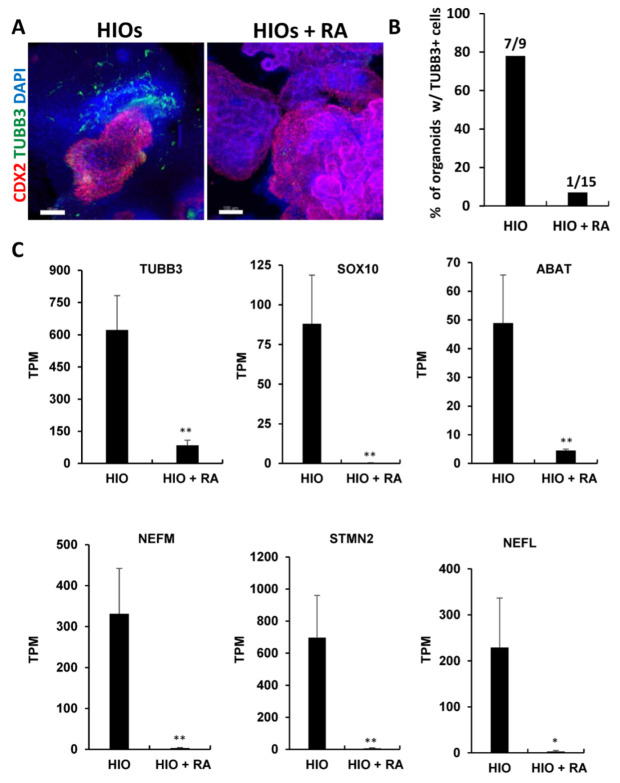
Retinoic acid inhibits the co-development of neurons in HIOs. (**A**) Whole-mount immunofluorescence staining of 35-day-old organoids from (HIOs and HIOs + RA) for CDX2(red), TUBB3 (green) and counterstained with DAPI. (**B**) The quantification of the percentage of organoids with TUBB3-positive cells. The number on the graph represents the number of organoids with TUBB4-positive cells divided by the total number of organoids analyzed. (**C**) Graphs of TPM values of neuronal genes from RNA-seq data of 35-day-old HIOs and HIOs + RA. Values are displayed as the mean ± SD. n = 4 per treatment group. * denotes a *p* value < 0.05. ** denotes a *p* value < 0.01 based on *t*-test.

Gene ontology analysis of RNA-seq data revealed that neuronal genes were inhibited in HIOs + RA compared to the controls (Table S2). To confirm these results, we performed wholemount immunofluorescence staining and quantification of the percentage of organoids stained positive for the neuronal marker Tubulin Beta Class III (TUBB3). This analysis revealed that HIOs contained co-developing neurons that resided in the organoid mesenchyme (Figure 6A). In contrast, TUBB3-expressing neurons were absent in HIOs + RA. Quantification of the percentage of organoids with TUBB3-expressing neurons revealed that 78% of HIOs contained TUBB3-positive cells. In contrast, only 7% of HIOs + RA contained TUBB3-positive cells (Figure 6B). To further confirm these results, we examined the mRNA levels of multiple neuron-specific genes including TUBB3, SRY-Box Transcription Factor 10 (SOX10), 4-Aminobutyrate Aminotransferase (ABAT), Neurofilament Medium Chain (NEFM), Stathmin 2 (STMN2) and Neurofilament Light Chain (NEFL). We found that RA treatment consistently inhibited the expression of neuronal genes (Figure 6C). In summation, these results indicate that RA inhibits the co-development of neurons in HIO cultures.

## 3. Discussion

HIOs hold tremendous promise not only for modeling human intestinal development and disease, but also as a source of autologous transplantation material. Proof-of-concept studies in mice have demonstrated that both biopsy-derived and human pluripotent stem-cell-derived organoids can be transplanted into the colon of recipient mice [21,30,31]. Despite their potential, there are multiple barriers that will need to be overcome in order to translate organoids to clinical practice. The inefficiency of HIO generation from human pluripotent stem cells makes their generation cost restrictive. Bioengineering approaches have been applied to HIO generation in order to select spheroids with increased organoid-forming efficiency [32]. In this study, we demonstrate that HIOs generated from clumps can grow and mature similar to spheroid-derived HIOs. In addition, we demonstrate that RA is sufficient to improve organoid-forming efficiency from clumps. These improvements in HIO generation should help improve efficiency and reduce the costs associated with differentiations that do not yield spheroids.

Regional specification imparts unique morphology and function to defined areas of the developing gastrointestinal (GI) tract. Although HIOs were previously shown to express PDX1, they rarely expressed GATA4 [5,6]. The lack of GATA4 expression in HIOs could explain why in vitro grown HIOs that are enriched in enteroendocrine cells, lack the expression of proximal small-intestine-specific hormones that are also lost in the intestine of mice when Gata4 was deleted from the intestinal epithelium [8]. We predict that RA will impart HIOs with the competence to generate GATA4-dependent enteroendocrine cell subtypes. Gata4 has also been shown to positively regulate the proliferation of epithelial cells in mid-gestation mouse embryos [33]. Future gain-of-function and loss-of-function studies could determine the role of GATA4 on the increased abundance of epithelial cells that we see in RA-treated HIOs.

The improved epithelial growth of RA-treated HIOs compared to control HIOs is a phenotype that we have observed consistently across HIOs from multiple IPSC lines. In addition, we also observe a severe reduction in the amount of mesenchyme in HIOs. Our analyses indicate that although RA-treated HIOs contain less mesenchyme, the mesenchyme is enriched for cell types known to stimulate the growth of intestinal epithelium. It is unclear if the increased organoid-forming efficiency and the increased epithelial composition of RA-treated HIOs is due to the direct effects of RA on the epithelium and/or direct effects of RA on the mesenchyme. RA may have a direct effect on epithelial growth through the induction of GATA4 which has been shown to be RA inducible [24]. Conversely, RA may act directly on the mesenchyme to inhibit its growth and select mesenchyme which expresses higher levels of RSPO3, which in turn will improve epithelial growth. Future single-cell RNA sequencing studies at various timepoints during differentiation should help elucidate the direct and indirect effects of RA. The use of RA could circumvent the need for niche factors allowing the cost-effective scale-up of organoid production. Furthermore, RA could be used to expand and isolate epithelium-supporting mesenchyme for the purposes of regenerative medicine.

The presence of neurons in HIOs is likely due to two factors: First, although the induction of mid/hindgut endoderm and mesoderm is highly efficient, a small percentage of the cells can be neural progenitors. Second, the addition of B27 and N2 supplements, which were originally intended for the enhancement of neural growth, likely stimulates the growth and differentiation of neural progenitors. Retinoic acid treatment resulted in a significant reduction in neuronal cells within HIOs. It is unclear if this is due to direct cytotoxicity of RA or if it is an indirect effect of the inhibition of mesenchyme growth. Future experiments will attempt to resolve these questions. In conclusion, our study highlights the benefits of RA treatment on HIO culture and should help elucidate mechanisms of epithelial growth, patterning and epithelial–mesenchymal signaling.

## 4. Materials and Methods

### 4.1. Cell Culture

Human-induced pluripotent stem cells IPSC72.3 (obtained from the Cincinnati Children’s Hospital Pluripotent Stem Cell Facility) and K3 (obtained from Medical University of South Carolina Cell Models Core) were grown in feeder-free conditions in six-well Nunclon^®^ surface plates (Thermo fisher Scientific, Waltham, MA, USA) coated with Matrigel (BD Biosciences) and maintained in mTeSR1 media (Stem Cell Technologies, Vancouver, Canada) at 37 °C with 5% CO_2_. Both of these lines have been previously characterized [34,35]. Cells were passaged every 4 days using Dispase solution (Thermo-Fisher Scientific) and were checked daily for differentiation. The cell line was checked for karyotype and routinely checked for mycoplasma.

### 4.2. Generation of Human Gut Tube Cultures

Human intestinal organoids (HIO) were generated and maintained as previously described [36,37]. For definitive endoderm (DE) induction, IPSCs were passaged with Accutase (Invitrogen, Waltham, MA, USA) and plated at a density of 100,000 cells per well in a Matrigel-coated, Nunclon^®^ surface 24-well plate (Thermo fisher Scientific, Waltham, MA, USA). On the first day of splitting, 10 μM Y27632 compound (Sigma) was added to the mTeSR1 media. After 24 h, media were changed to mTeSR1 and cells were grown for an additional 24 h. Then, the cells were treated with 100 ng/mL of Activin A for 3 days as previously described for DE formation [6]. DE was then treated with hindgut induction medium (RPMI 1640, 2 mM L-glutamine, 2% decomplemented FBS, penicillin-streptomycin and 100 ng/mL Activin A) for 4 days with 500 ng/mL FGF4 (R&D) and 3 μM Chiron 99021 (Tocris) to induce the formation of mid/hindgut floating spheroids and mid/hindgut endoderm which remained in the monolayer.

### 4.3. The Culture of Floating Spheroids and Clumps of Dissociated Mid/Hindgut Endoderm

Floating spheroids were collected from 24-well plates and plated in Matrigel (BD) with a minimum of 30 spheroids plated per well. For the generation of clumps from the mid/hindgut endoderm monolayer, 1000 µL pipet tips were used to scratch the monolayer generating clumps that detached from the plate. Clumps floating in media were then transferred to 15 mL tubes. Clumps were further triturated by pipetting up and down using a 1000 µL pipet to allow for filtering through a 200 μm cell strainer (Pluriselect, Leipzig, Germany). Clumps we passed through the 200 μm cell strainer and the flow through clumps were plated in Matrigel with a minimum of 100 clumps plated per well in 24-well plate. To generate proximal HIOs, spheroids or clumps were overlayed with intestinal growth medium (Advanced DMEM/F-12, N2, B27, 15 mM HEPES, 2 mM L-glutamine, penicillin-streptomycin) supplemented with 100 ng/mL EGF (R&D) alone, or 100 ng/mL EGF with 2 μM RA (R&D). Media were changed twice weekly thereafter. HIOs and HIOs + RA were replated in fresh Matrigel every 14 days at a density of 5–10 organoids per well and kept in culture until day 35.

### 4.4. Efficiency of Organoid Formation

The filtered broken clumps were plated in Matrigel with a maximum of 100 clumps plated per well of a 24-well plate. The number of clumps per well was counted on day 7. After culturing in intestinal growth medium with EGF or EGF + RA for 2 weeks, the number of organoids per well was counted for 21-day-old organoids. We then calculated the percentage of organoid formation by dividing the number of 21-day-old organoids by the number of day 7 clumps, multiplied by 100. These experiments were repeated 3 times.

### 4.5. Immunofluorescence Staining

Organoids were fixed in 4% PFA, washed in PBS and then placed in 30% sucrose overnight at 4 °C on a rocking platform. Organoids were then frozen in OCT. After cryosection, the slides with tissues were blocked using normal donkey serum (5% serum in PBS plus 0.5% Triton-X) for 30 min at room temperature. Then, the tissues were incubated with primary antibody overnight at 4 °C. Next day, slides were washed 3x with 1x PBS plus 0.5% Triton-X (PBST) and incubated in secondary antibody with DAPI in blocking buffer for 2 h at room temperature. Please see Table S3 for a list of antibodies and respective dilutions. Slides were then washed 2X with PBST followed by a final wash in 1x PBS. Coverslips were then mounted using Fluoromount-G^®^ (SouthernBiotech, Birmingham, AL, USA). Images were captured on a Zeiss LSM 880 NLO with Airscan confocal microscope and analyzed using Imaris Imaging Software 9.9 (Oxford Instruments, Abingdon, UK).

For whole-mount staining, PFA fixed organoids were washed with PBS and then permeabilized in PBST overnight at 4 °C on a rocking platform. Organoids were blocked for 6–8 h at room temperature on a rocking platform, then incubated in primary antibody overnight at 4 °C on a rocking platform. Next day, the organoids were washed 3–5x with PBST and incubated in secondary antibody overnight at 4 °C on a rocking platform. Then, the organoids were washed 2x with PBST followed by a final wash in PBS. Organoids were dehydrated by washing them 3 times in 100% Methanol and cleared using Murray’s clear (2 parts benzyl benzoate and 1 part benzyl alcohol). Images were captured on a Zeiss LSM 880 NLO with Airscan confocal microscope and analyzed using Imaris Imaging Software 9.9 (Oxford Instruments, Abingdon, UK).

### 4.6. Quantification of Immunofluorescence Images

Image quantification of organoids was carried out on sections from which images were captured as explained above. The number positive nuclei for each marker were quantified using the spot function in Imaris.

### 4.7. Western Blot

The 35-day-old organoids were rinsed with ice-cold phosphate-buffered saline (PBS) and lysed with Triton buffer (40 mM HEPES pH 7.4, 150 mM NaCl, 2.5 mM MgCl_2_, and 1% Triton) supplemented with protease inhibitor (Thermo fisher Scientific, Waltham, MA, USA. Cat# A32953) and phosphatase inhibitors (phosphatase inhibitor cocktail sets I and II, Calbiochem). The cell lysates were centrifuged at 13,200  r.p.m. for 10 min at 4 °C. Equal amounts of protein were separated by SDS-PAGE, transferred to a polyvinylidene difluoride membrane (MilliporeSigma, Burlington, MA, USA), immunoblotted with antibodies and visualized with horseradish-peroxidase-coupled secondary antibodies.

### 4.8. RNA-Seq Processing

RNA was purified using Nucleospin^®^ RNA extraction kit (Macharey-Nagel, Düren, Germany) and sent to BGI for RNA library construction and RNA sequencing using the DNBseq platform. For sequencing data analysis, HIOs and HIO + RA data were analyzed by BGI. The sequencing reads were filtered using BGI internal software SOAPnuke to obtain clean reads which were stored in FASTQ format. The clean reads were mapped to the human genome with HISAT2 and Bowtie2. Gene expression level was calculated with RSEM. DEseq2 was used to obtain differentially expressed gene data.

For clump and spheroid data from Figure 1, Partek Flow software was used for data analysis. Analysis was initiated from FASTQ files, and clean reads were mapped to the human genome with STAR and quantified to annotation model using Partek E/M. DEseq2 was used to obtain differentially expressed gene data. PCA was conducted using Partek Flow for both RNA-seq data sets.

### 4.9. Quantification, Statistical Analysis and Data Availability

For RNA-seq experiments involving spheroids, clumps and in vitro grown organoids, “*n*” represents the number of biological replicates (2–3 wells were collected for each replicate). Quantified data are represented as mean ± SEM or mean ± and is noted in the figure legend. The “*n*” is also noted in the figure legends. To directly compare HIOs and HIOs + RA, *t* tests with 2-tailed distribution and two-sample equal variance were run in Microsoft Excel. RNA-seq data generated in this paper will be uploaded to GEO.

## Data Availability

Primary RNA-sequencing files will be deposited to GEO.

## Supplementary Materials

The following supporting information can be downloaded at: https://www.mdpi.com/article/10.3390/ijms23158624/s1.
